# Research waste in diagnostic trials: a methods review evaluating the reporting of test-treatment interventions

**DOI:** 10.1186/s12874-016-0286-0

**Published:** 2017-02-24

**Authors:** Lavinia Ferrante di Ruffano, Jacqueline Dinnes, Sian Taylor-Phillips, Clare Davenport, Chris Hyde, Jonathan J. Deeks

**Affiliations:** 1 Biostatistics, Evidence Synthesis and Test Evaluation Research Group, Institute of Applied Health Research, University of Birmingham, Birmingham, B15 2TT UK; 2 Warwick Medical School, The University of Warwick, Coventry, CV4 7AL UK; 3 PenTAG, The Institute of Health Reseach, University of Exeter Medical School, Exeter, EX1 2LU UK

**Keywords:** RCT, Test-treatment, Test Evaluation, Diagnostic accuracy, Patient outcomes, Reporting quality, Intervention reporting

## Abstract

**Background:**

The most rigorous method for evaluating the effectiveness of diagnostic tests is through randomised trials that compare test-treatment interventions: complex interventions comprising episodes of testing, decision-making and treatment. The multi-staged nature of these interventions, combined with the need to relay diagnostic decision-making and treatment planning, has led researchers to hypothesise that test-treatment strategies may be very challenging to document. However, no reviews have yet examined the reporting quality of interventions used in test-treatment RCTs. In this study we evaluate the completeness of intervention descriptions in a systematically identified cohort of test-treatment RCTs.

**Methods:**

We ascertained all test-treatment RCTs published 2004–2007, indexed in CENTRAL. Included trials randomized patients to diagnostic tests and measured patient outcomes after treatment. Two raters examined the completeness of test-treatment intervention descriptions in four components: 1) the test, 2) diagnostic decision-making, 3) management decision-making, 4) treatments.

**Results:**

One hundred and three trials compared 105 control with 119 experimental interventions, most commonly in cardiovascular medicine (35, 34%), obstetrics and gynecology (17%), gastroenterology (14%) or orthopedics (10%). A broad range of tests were evaluated, including imaging (50, 42%), biochemical assays (21%) and clinical assessment (12%). Only five (5%) trials detailed all four components of experimental and control interventions, none of which also provided a complete care pathway diagram. Experimental arms were missing descriptions of tests, diagnostic-decision making, management planning and treatments (36%, 51%, 55% and 79% of trials respectively); control arms were missing the same details in 61%, 66%, 67% and 84% of trials.

**Conclusion:**

Reporting of test-treatment interventions is very poor, inadequate for understanding the results of these trials, and for comparing or translating results into clinical practice. Reporting needs to improve, with greater emphasis on describing the decision-making components of care pathways in both pragmatic and explanatory trials.

Please see the companion paper to this article: http://bmcmedresmethodol.biomedcentral.com/articles/10.1186/s12874-016-0287-z.

## Background

Healthcare interventions need to be described with adequate detail in reports of randomized controlled trials (RCTs) to allow clinicians and other decision-makers to implement effective interventions reliably [1], whilst enabling researchers to understand why an intervention may not have been successful. Inadequate intervention descriptions also hinder our ability to examine the validity of trials, and accurately determine their eligibility for inclusion in systematic reviews. Yet a growing number of reviews find that interventions are often very poorly described [2–7].

Guidelines detailing reporting standards for protocols and final reports of RCTs provide some guidance on describing study interventions. Adequate reporting of trial interventions has been widely encouraged since 2001 [8], the most recent guidance from CONSORT [9] (Consolidated Standards of Reporting of Trials, item 5) and SPIRIT [10] (Standard Protocol Items: Recommendations for Interventional Trials, item 11) recommends that trial protocols and reports should describe “interventions for each group with sufficient detail to allow replication, including how and when they will be administered”. Most recently, the TIDieR initiative (Template for Intervention Description and Replication) has introduced a checklist extension to SPIRIT and CONSORT to help researchers to more fully describe interventions, with the ultimate aim of improving their replicability [11].

Complex interventions have the added challenge of multiple interacting components, each of which needs to be described in adequate detail to allow replication. Some fields, such as physiotherapy and psychology, document standardized interventions in treatment manuals, however, a recent review of 137 nonpharmacologic RCTs found that such materials are infrequently (47%) published [4]. Studies comparing the reporting quality of pharmaceutical with nonpharmacologic interventions have concluded that complex interventions are particularly difficult to describe, standardize, reproduce and administer consistently [2, 6, 12].

Randomized trials evaluating the effects of diagnostic tests on patient outcomes are widely heralded as the most rigorous method for evaluating the effectiveness of tests [13–15]. They evaluate complex interventions comprising steps of testing, decision-making and treatment. Notably, each patient does not receive the same treatment: rather the treatment choice is personalized by decision-making incorporating the results of the tests obtained in the first step with existing diagnostic information, additional testing, and the preferences and values of patients and clinicians, all within the context of a particular health setting. Test-treat interventions can be evaluated in various study designs [16]. RCTs are not always necessary, and are often expensive and difficult to undertake. Our companion review of the methodological quality of the trials in this cohort indicates that they suffer inherent methodological challenges and are often at risk of bias due to poor methodological quality [17].

**Fig. 1:**
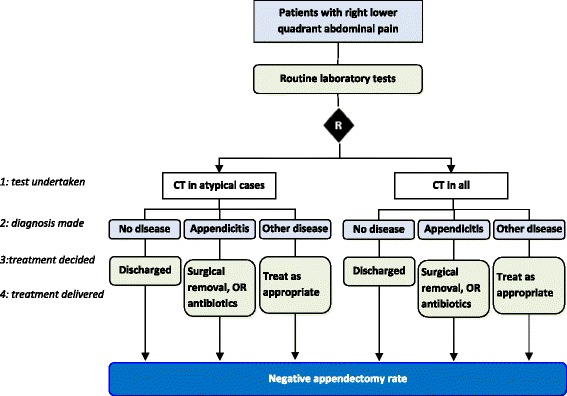
The four steps of a test-treatment strategy using abdominal CT to diagnose appendicitis in patients presenting with right lower quadrant abdominal pain. The trial compared two diagnostic strategies for confirming or ruling out suspected acute appendicitis in adults presenting to the emergency department with right lower quadrant abdominal pain [18]. The routine approach of scanning all such patients with CT was compared with a new strategy of ordering CT only when indicated by specific signs and symptoms. In both arms the 4 test-treatment steps consist of testing (routine laboratory tests +/− a CT scan), a diagnostic decision (appendicitis, other disease, no disease), a management plan for each group (surgery or antibiotics to treat appendicitis, discharge for disease free patients, and treatment as necessary for alternative conditions), and delivery of these treatments. The trial’s primary outcome measured the proportion of removed appendices that were disease-free

A description of a test-treatment strategy thus involves four main steps: 1) a test is undertaken; 2) the test result is used (often with other information) to place patients into diagnostic categories; 3) a management plan is determined for patients in each category; 4) the treatments are delivered. For example, a trial may investigate whether abdominal computed tomography (CT) in patients presenting with suspected acute appendicitis improves patient outcomes (Fig. 1) [18]. The complex intervention being trialed includes: 1) a CT scan; 2) categorizing each patient according to features observed alongside existing diagnostic information into different diagnostic groups (such as highly likely appendicitis, possibly appendicitis, urinary tract infection, gastroenteritis, or ectopic pregnancy); 3) making a management plan for patients in each group (such as immediate surgery, watch and wait, antibiotics); 4) delivering the treatments.

Diagnostic and therapeutic decision-making are renowned for being highly variable: the interpretation of test results is likely to differ according to expertise [19, 20], clinicians have individual attitudes to the balance of risks resulting from missing diagnoses or over-treating patients [21], while patients have highly variable attitudes and perceptions toward their management [22, 23]. Small-scale observations have reported a common lack of intervention detail, particularly for the decision-making elements of test-treatment strategies [24–26]. Consequently, researchers have hypothesized that test-treatment strategies may be very challenging to document [16, 25, 27, 28]. However, no reviews have yet examined the reporting quality of interventions used in test-treatment RCTs.

In this study we evaluate the quality of reporting of test-treatment interventions in a systematically identified cohort of trials of diagnostic tests to determine reporting rates for each component of the experimental and control interventions.

## Methods

### Search

We searched the Cochrane Central Register of Controlled Trials (CENTRAL, Issue 2 2009) for test-treatment RCTs that were published between 2004 and 2007 using a validated, previously published strategy [29] developed by information specialists comprising general diagnostic and methodology terms (Table 1). CENTRAL was used because it includes all reports of RCTs indexed as study types in MEDLINE and identified from systematic, sensitive searches of both MEDLINE and EMBASE, additional hand-searched material, and other extensive database searches contributed through the Cochrane specialized registers.

**Table 1:** Search strategy for test-treatment RCTs

Search strategy		Hits
#1	Sensitiv^a^ or diagnose or diagnosis or diagnostic^a^ in Clinical Trials	70,052
#2	Random^a^ in Clinical Trials	335,175
#3	“Study design” next “rct” in Clinical Trials	150,275
#4	(#2 OR #3)	449,453
#5	(#1 AND #4)	50,419
#6	(#5), from 2004 to 2007	12,892

CENTRAL Issue 2 2009 (Wiley InterScience, searched 29 May 2009)–general diagnosis textwords across all fields limited to publication years 2004 to 2007

^a^Denotes truncation of search term

### Study selection

Eligible trials randomized patients between diagnostic testing strategies and measured at least one patient outcome occurring after treatment. We excluded trials evaluating tests for screening and monitoring, and non-English language studies. Duplicate records were identified using title-based sorting in Microsoft Access, and during full-text screening. Multiple publications relating to a single trial were assimilated through cross-referencing, with all material used for analysis. Titles and abstracts of potentially relevant studies were screened by one author (LFR) and full-text reports of relevant abstracts reviewed to determine inclusion. A random 10% sample of citations was screened independently by a second author (CD) to measure the reliability of the screening process [29]. Discrepancies in the relevance of full articles were discussed and resolved by consensus.

### Data extraction and analysis

Trials were classified by journal type (general medical journals, publishing on any medical topic, vs specialist journals), trial design, clinical specialty and care setting. Test-treatment strategies were classified according to the diagnostic technologies evaluated and the type of diagnostic comparison, namely whether a new test was compared to an existing test, (‘replacement’ comparison), used to filter which patients should receive an existing test, (‘triage’ comparison), or added to an existing testing strategy (‘add-on’ comparison). Experimental study groups were defined by specific reference to a new test to be introduced under evaluation. Control arms were identified when referred to as the comparator, current care standard or common clinical practice. In a minority of cases (7/103, 7%), the control arm was identified implicitly by the manner in which study results were discussed.

We appraised written descriptions of intended test-treatment strategies in four components. We defined these components using published frameworks that outline which essential elements of test-treat pathways lead to changes in patient health. These commonly present three steps: information, decision, and action [30, 31], which recently have been extended [23] to fully separate the process of diagnosis from that of subsequent treatment. The resulting four components are: 1) The Test: what diagnostic test was given and how was it performed? 2) Diagnostic decision-making: how was the output from the test categorized and used to define diagnostic groups? 3) Management decision-making: how was each diagnostic group managed? 4) The Treatment: what interventions were given and how?

Reporting of each component was assessed as present or absent. A component was judged as reported if any description was provided or cited in any publications reporting on a given trial. This included related publications not identified by the project search (e.g., design protocols, original trial reports, long-term follow-up papers), that were traced through citations and author-title searches of Medline. We also systematically retrieved the full text of cited descriptions to check whether citations were relevant. We did not require descriptions to be sufficient to allow the replication of interventions, as required by CONSORT or TIDier; therefore while our assessments are objective, they will overestimate the proportion of trials complying with CONSORT or TIDieR. Components 1) and 4) were judged as reported if the name of the test (component 1) or treatment (component 4) were given plus any one additional piece of relevant information (for example the threshold for a test, or a dose for a drug); articles providing only the name of the test or treatment were considered as inadequately reported. Components 2) and 3) were judged as present if any information was given on decision-making (such as how particular test results confer a particular diagnosis, or how treatments were selected for different diagnoses). Where trials contained a ‘no testing’ arm (for example, comparing test driven therapy against empirical therapy) only item 4) was recorded, items 1)-3) were coded as not applicable. The numbers of reported items were compared across test types and care settings.

Reports were also appraised for their use of diagrams to explain the care pathway algorithms, following good practice recommendations by the MRC for the evaluation of complex interventions [32]. Diagrams were considered complete if they conveyed all four test-treatment components for each trial arm, and partially reported if at least one component was not represented in any trial arm.

Extraction and quality assessment was performed independently in duplicate by four authors (LFR, JD, STP, JJD). Findings are described using percentages.

## Results

**Fig. 2:**
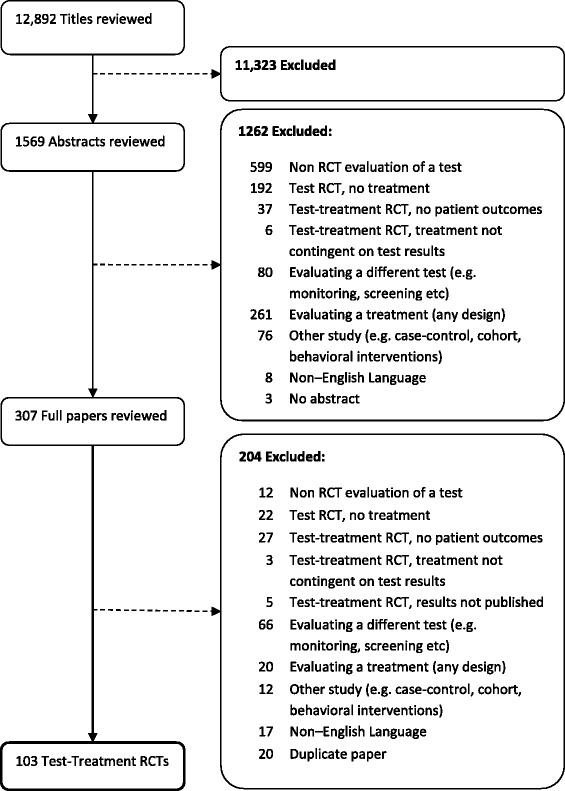
Identification of test-treatment RCTs from CENTRAL, searched 2009, Issue 2

The search strategy retrieved 12,892 citations (Table 1), of which 1401 were screened in duplicate. Agreement was substantial (k = 0.74), and full concordance results have previously been published [29]. The final cohort contained 103 trials comparing 105 control interventions with 119 experimental interventions (Fig. 2).

### Characteristics of trials

Trial reports were published in 70 journals, with only 21 journals publishing more than two. Most frequent journals were *Radiology* (*n* = 5), *Health Technology Assessment* (*n* = 4), *New England Journal of Medicine* (*n* = 4), *Human Reproduction* (*n* = 4), *American Journal of Obstetrics and Gynecology* (*n* = 3), *Journal of the American College of Cardiology* (*n* = 3), and *Ultrasound in Obstetrics and Gynecology* (*n* = 3). Overall 66% (*n* = 68) were published in specialist journals. Characteristics of the included studies are summarized in Tables 2 and 3. A broad range of technologies were evaluated across 18 specialties, with imaging the subject of evaluation in almost half the trials. Some trials compared testing to no testing by evaluating the benefits of removing testing from a previously tested population (experimental ‘no test’ comparisons), or of introducing a new test in a previously untested population (control ‘no test’ comparisons).

**Table 2:** Characteristics of included trials

Trial characteristics	Total (*N* = 103)		2004–5 (*N* = 56)		2006–7 (*N* = 47)	
	n	(%)	n	(%)	n	(%)
Trial design:						
Parallel	90	(87)	49	(89)	41	(87)
Factorial	3	(3)	2	(4)	1	(2)
Cross-over	1	(1)	0	(−)	1	(2)
Randomized disclosure	9	(9)	5	(7)	4	(9)
Randomization:						
Individual	97	(94)	53	(96)	44	(92)
Cluster	6	(6)	2	(4)	4	(8)
Number of study arms:						
2	92	(89)	50	(91)	42	(88)
> 2	11	(11)	5	(9)	6	(13)
Diagnostic comparison:						
Triage	17	(17)	9	(16)	8	(17)
Additional	28	(27)	15	(27)	13	(27)
Replacement	60	(58)	32	(57)	28	(58)
Medical Specialty:						
Cardiovascular Medicine	35	(34)	20	(36)	15	(31)
Embryology	2	(2)	0	(−)	2	(4)
Emergency Medicine	1	(1)	0	(−)	1	(2)
Gastroenterology	14	(14)	6	(11)	8	(17)
General Medicine	1	(1)	0	(−)	1	(2)
Geriatrics	1	(1)	1	(2)	0	(−)
Infectious diseases	1	(1)	1	(2)	0	(−)
Neurology	3	(3)	1	(2)	2	(4)
Obstetrics and Gynecology	17	(17)	6	(11)	11	(23)
Oncology	5	(5)	5	(9)	0	(−)
Ophthalmology	2	(2)	2	(4)	0	(−)
Orthopedics	10	(10)	8	(15)	2	(4)
Otolaryngology	1	(1)	1	(2)	0	(−)
Psychiatry	2	(2)	0	(−)	2	(4)
Respiratory	3	(3)	1	(2)	2	(4)
Urology	2	(2)	1	(2)	1	(2)
Multiple	3	(3)	3	(5)	0	(−)

**Table 3:** Test types evaluated in the 103 test-treatment RCTs

Test type	Control	(%)	Experimental	(%)
Biochemical	10	(10)	25	(21)
Biopsy	1	(1)	2	(2)
Clinical	18	(17)	14	(12)
Electrophysiology	7	(7)	10	(8)
Imaging (all)	35	(33)	50	(42)
*Endoscopy*	*14*	*(13)*	*11*	*(9)*
*Radiology*	*21*	*(20)*	*39*	*(33)*
Telemedicine	2	(2)	2	(2)
Standard Care	10	(10)	0	(0)
Multiple test interventions	8	(8)	12	(10)
No test	14	(13)	4	(3)
Total	^a^105		^a^119	

^a^denominators refer to the number of study arms

### Reporting of interventions

Trialists used between 34 and 1305 words (median: 336, IQR: 153–560) to describe test-treatment interventions. Descriptions of the clinical processes involved were often poor, characterized by very low levels of detail and frequently failing to report several test-treatment components. Examples of well reported descriptions and our judgements are provided in Table 4 [32–36].

**Table 4:** Definition of the four components used to assess the description of test-treatment interventions, with examples of adequate descriptions

Component	Definition and example
1. Diagnostic test:	Technique used to perform the test. Reporting the name of the test only was considered insufficient.
	e.g., *“Radiographs of the knee were obtained in the lateral and anteroposterior projection and were supplemented with patellar or tunnel views if pathologic abnormalities of the patellofemoral joint or intercondylar notch were suspected”* [33].
2. Diagnostic decision:	Description of the operational criteria used for arriving at a particular diagnosis using the test results.
	e.g., *“If the lung scan showed no abnormalities, pulmonary embolism was excluded; if there were 1 or more segmental perfusion defects that were normally ventilated, the scan was considered diagnostic for pulmonary embolism (“high-probability scan”); and if there were perfusion defects that did not meet criteria for a “high-probability scan,” the scan was considered nondiagnostic”* [34].
3. Management decision:	Description of how treatments were selected as a result of the diagnosis.
	e.g., *“Patients in group A1 and group A2 with H. pylori sensitive to AMO and LEV were treated with AMO (1 g b.i.d.), LEV (500 mg b.i.d.), and ESO (esomeprazole) (20 mg b.i.d.) for 10 days. If H. pylori was found resistant to AMO and/or LEV the treatment was based on the indications of the susceptibility test. Patients enrolled in group B1 and group B2 were treated empirically, that is without performing the H. pylori susceptibility to various antibiotics, with a standard treatment that included AMO (1 g b.i.d.), LEV (500 mg b.i.d.), and ESO (20 mg b.i.d.) for 10 days”* [35].
4. Treatment:	Description of how selected treatments were administered. Reporting of the treatment name only was considered insufficient.
	e.g., *“After ultrasound diagnosis of an anal sphincter tear… women were brought immediately to the operating room to provide appropriate lighting, instruments, and assistants and underwent a surgical exploration of the perineum by the obstetrician-in-charge under senior supervision. The anal sphincter was exposed and its integrity assessed by inspection and palpation. The ends of the sphincter were approximated end-to-end with 2–0 monofilament polyglyconate sutures (Maxon, Sherwood Davis & Geck, St. Louis, MO). Postoperatively, women received dietary advice to avoid constipation, with occasional use of stool softeners. For women allocated to the control group, the obstetrician sutured the perineum after clinical examination”* [36].

**Fig. 3:**
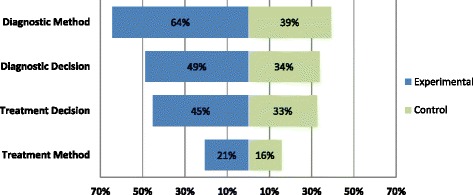
Proportion of trial arms describing each element of the test-treatment protocol. Some strategies did not require description of a test or treatment (or associated decision-making), for example when evaluating the addition of a new test to no test, or the removal of treatment. Denominators differed when test-treat strategies did not involve a test or a treatment (e.g., trials comparing the benefits of treating all vs. testing to select those for treatment [45]). The denominators for frequency calculations were reduced accordingly: Diagnostic method: Experimental *n* = 115, Control *n* = 92. Diagnostic decision: Experimental *n* = 115, Control *n* = 92. Treatment decision: Experimental *n* = 115, Control *n* = 95. Treatment method: Experimental *n* = 117, Control *n* = 102

Tests were the most commonly described component, reported for over half of experimental strategies (64%, 74/115) though for little more than a third of control groups (39%, 36/92) (Fig. 3). Diagnostic decision-making criteria were mentioned in 49% (56/115) of experimental and 34% (31/92) of control strategies, while management decisions were mentioned in 45% (52/115) of experimental and 33% (31/95) of control strategies. Treatment interventions were the least frequently reported element, outlined in only 21% (24/117) of experimental strategies, and 16% (16/102) of control strategies.

All 4 components were missing from at least one arm of 43 trials (42%), including 13 trials (13%) for which no information was presented for any arm. Trials were twice as likely to omit all description for control interventions (36%) than for experimental interventions (18%) (Table 5). Thirteen percent (13/103) described all four components of experimental interventions, and 11% (11/103) did so for control interventions. Only 5 of the 103 RCTs (5%) described all four test-treat components for both control and experimental study arms.

**Table 5:** Reporting of control vs. experimental interventions

Number of components reported	^a^Control interventions		^a^Experimental interventions		^b^Control arm per trial		^b^Experimental arm per trial	
	n	(%)	n	(%)	n	(%)	n	(%)
0	38	(36)	24	(20)	37	(36)	19	(18)
1	19	(18)	25	(21)	19	(18)	22	(21)
2	21	(20)	29	(24)	20	(19)	26	(25)
3	16	(15)	27	(23)	16	(16)	23	(22)
4	11	(10)	14	(12)	11	(11)	13	(13)
Total	105	(100)	119	(100)	103	(100)	103	(100)

Table presents counts of the number of individual interventions (*n* = 224) and total number of included trials (*N* = 103) that reported between zero and all four components of test-treatment interventions. Items deemed not applicable were counted as reported

^a^Number of study arms reporting 0–4 components

^b^Number of trials reporting 0–4 components in at least one study arm (some trials evaluated multiple control and/or experimental groups)

Reports evaluating electrophysiological tests (e.g., cardiotocography), clinical examinations and telemedical consultations described the fewest components. Trials that described control arms as ‘standard care’ reported few of the required elements (Table 6). Reporting was worse for test-treatment strategies conducted across multiple care settings, though no difference was observed between individual care sectors (Table 6). Trials evaluating replacement testing strategies were less likely to report at least 2 components (31/60, 52%) when compared to those evaluating add-on (19/28, 68%) or triage comparisons (13/17, 76%).

**Table 6:** Reporting of control vs. experimental interventions

Trial characteristic	Control (*N* = 105)		Experimental (*N* = 119)		Total (*N* = 224)	
	>2 components reported	(%)	>2 components reported	(%)	>2 components reported	(%)
Test type:						
Biochemical	3	(30)	10	(40)	13	(37)
Biopsy	0	(−)	1	(50)	1	(33)
Clinical	2	(11)	6	(43)	8	(25)
Electrophysiology	2	(29)	1	(10)	3	(18)
Imaging (all)	6	(17)	17	(34)	23	(27)
*Endoscopy*	*2*	*(14)*	*5*	*(45)*	*7*	*(28)*
*Radiology*	*4*	*(19)*	*12*	*(31)*	*16*	*(27)*
No test	12	(86)	4	(100)	16	(89)
Standard Care	1	(10)	0	(−)	1	(10)
Telemedicine	0	(−)	0	(−)	0	(−)
Multiple	2	(25)	2	(17)	4	(20)
Care sector:						
Emergency	3	(25)	5	(42)	8	(33)
Primary	4	(36)	10	(56)	14	(48)
Secondary	9	(20)	12	(24)	21	(22)
Tertiary	12	(43)	13	(43)	25	(43)
Multiple	0	(−)	1	(13)	1	(6)
Total	28	(27)	41	(34)	69	(31)

Table presents counts of the number of individual interventions for which 3 or more test-treatment components were reported

### Use of care-pathway diagrams

Diagrams were included by approximately one-fifth of trials, summarizing 20% (24/119) of experimental interventions and 21% of (22/105) control interventions. Care pathway diagrams were most informative when they illustrated how patient management decisions depended on the diagnostic findings. Fewer than 10% (8 experimental, 7 control) provided this information.

## Discussion

### Key findings

In 98 (95%) of the 103 test-treatment RCTs, descriptions of interventions did not mention all the components necessary to characterize test-treatment strategies. Only five trials (5%) provided a description of tests and test methods, treatment methods and decision-making across all study groups, none of which also provided a complete care pathway diagram. We noted that test-treatment interventions for control groups were particularly poorly reported. Descriptions of experimental interventions most often provided details of tests, but less than half gave details of diagnostic and management decision-making, and less than a quarter mentioned which treatments were subsequently used.

In many of these circumstances, failure to describe the test-treatment interventions will make it impossible for clinicians to assess whether the trial is applicable to their practice, nor be able to implement the test-treatment intervention should they choose. The potential implications of such poor reporting is that the time and resources invested in these trials may be largely wasted [37].

### Interpretation of findings

Our study is the first to analyze the reporting quality of interventions in RCTs of test-treatment strategies. Other studies evaluating the reporting quality of complex interventions have noted inadequate descriptions of interventions in 87% of back-pain RCTs [38], 59% of surgical treatment trials [5] and 61% of non-pharmacological intervention RCTs [4]. Our finding that test-treatment strategies are inadequately described by 95% of trials suggests that there are additional challenges to describing these interventions. We present three explanations for our findings.

First, many trials have used an approach that solely specifies a new test in the study arm against a control arm of standard care, without providing any further specification of the test-treatment intervention in either arm. Overall 24% (25/103) of trials reported only the tests used in the different arms, while 8 of the 10 trials comparing new tests against a control arm of standard care failed to specify the tests or any subsequent details of management and treatment in the control arm. For example, a trial of routine lumbar X-ray in patients with acute lower back pain (LBP) simply described the interventions as: “In addition to receiving the usual care for patients with LBP, the intervention group patients had lumbar spine radiography at the baseline interview. The control group received the usual care without lumbar spine radiography” [39]. These trials typically neither specified nor recorded how test results were used for decision-making, what pathologies were diagnosed, or what treatments were actually used.

Secondly, test-treatment pathways may be difficult to describe, or even enumerate, due to the myriad of possible downstream actions following a test. We know from trials of complex therapeutic interventions that decision-making processes are very difficult to circumscribe into standardized, rigid protocols, and these problems could account for the poor descriptions we observed of clinical examinations, tele-medical consultations and multiple-test interventions (Table 5). On the other hand we found complex endoscopic techniques were often standardized and well described, despite often being part of multistage diagnostic processes.

Finally, the link between better reporting of experimental and control interventions in a minority of studies suggests there is a lack of awareness amongst trialists and investigators of the level of detail which needs to be included in a trial report. Journal instructions to authors of trial reports have been found lacking, with only 14% (15/106) providing specific directives regarding the reporting of interventions [40]. Thus the need to describe several components of multistage complex interventions is likely to be more poorly recognized.

### Requirements for pragmatic RCTs of test-treatment interventions

There is general acceptance that results of pragmatic trials are more applicable to standard practice than explanatory trials [41]. We did not formally assess the position of our trials on the pragmatic explanatory continuum [42], however all the studies we examined were undertaken in the ‘real world’ and evaluated the impact of new testing strategies, alongside current practice, on patient health. We would argue that the notion of ‘explanatory’ trials does not apply well to test-treatment RCTs seeking to evaluate downstream health consequences, but rather best describes studies such as those evaluating diagnostic accuracy (‘does the new test correctly discriminate between diseased and non-diseased patients?’). For a test-treatment RCT, undertaking a pragmatic approach involves recruiting patients as they present in standard care, utilizing tests and treatments as would be provided in the health service, and allowing flexibility to tailor interventions to individual patients as would occur in practice (including allowance of non-compliance, cross-overs and drop-outs). Nevertheless, guidance for pragmatic trials clearly states that the intended interventions in all arms should be defined precisely [43, 44].

Findings from a new test are most likely to be used in an effective way if information on their diagnostic value, and guidance on how they should impact on management, are provided. This is particularly important for new test technologies, where clinicians may be unsure about basing management decisions on new diagnostic information and, in the absence of guidance, could ignore results of the new test or respond to it in inconsistent ways; both would bias studies towards finding no difference.

The absence of complete description of the interventions in the majority of studies might have arisen from poor reporting, but also because the trial protocols may never have specified how test results were to be interpreted and used to determine treatment. Such an approach could have arisen if trialists wrongly considered it to be appropriate in a pragmatic trial design. Other possible explanations include challenges in documenting the components in the test-treatment intervention; concern that specifying a particular care pathway may limit recruitment; or in some circumstances uncertainty around how the results of a test could be best used to determine treatment. Greater preparatory work to fully develop and specify the test-treatment intervention, and obtain buy-in from clinicians involved in the trial, might solve these issues. An RCT of a test strategy that commences before it is determined how test results should be used could ultimately reflect variation in clinician behavior more than the potential value of a diagnostic technology.

There are arguments against specifying control test-treatment strategies in trials whose explicit purpose is to compare outcomes from organized diagnostic services with unstructured care (14 examples were found in our cohort). Since this comparison is of a formalized diagnostic strategy against an approach allowing a clinician to operate without guidance, it is clear that introducing a protocol for decision-making in the control arm would eliminate any effect.

Good pragmatic trials of test-treatment interventions should also report the diagnoses made and treatments undertaken in each arm of the trial, delineated by test results so that one can assess the degree of adherence to the recommended test-treatment protocols. Whilst several studies reported measures of diagnostic and therapeutic impact aggregated in each trial arm, it was very rare for this to be presented according to the test result.

### Recommendations

**Fig. 4:**
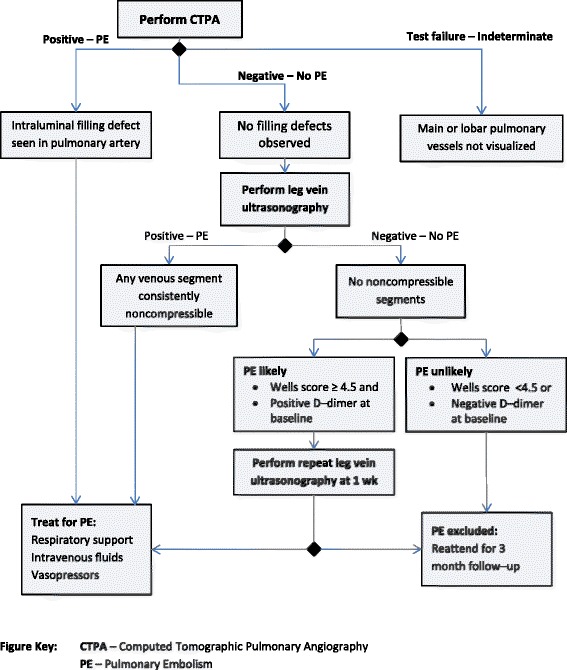
Example of a decision-tree graphic summarizing the 4 key components of one test-treatment intervention. Developed to illustrate an intervention evaluated in a published RCT [46]

We make three recommendations. First, reporting can be improved by providing guidance for authors. The TIDieR checklist provides a useful tool to assist in describing interventions, but does not explicitly consider complex “staged” interventions such as test-treatment trials. These require further development of Item 9 in TIDieR (handling the tailoring of interventions that are not identical for all participants, which requires descriptions of “how, why, what and when interventions are personalized, titrated or adapted”) [11]. The current tool does not highlight that this should include a full delineation of all management pathways according to test results in test-treatment comparisons. These might best be summarized graphically in a decision-tree which outlines the different sets of test results, the diagnoses which can be made from them, and the possible actions which could occur at each step, as illustrated in Fig. 4. In future, additional detail might be provided in ‘diagnostic intervention manuals’, mirroring complex intervention practice, although such an approach should first be investigated to ensure it provides a useful addition to intervention description.

Second, all trials must aim to prescribe the diagnostic strategies and care pathways that should be followed and describe them in adequate detail to allow replication. Whilst trials evaluating highly flexible interventions allow deviation from planned care pathways as clinically required, the pathways should be specified with as much detail as possible in the study protocol and report. For highly variable diagnostic strategies that are difficult to translate into a prescriptive format, such as clinical consultations, trialists should as a minimum aim to standardize the intended function of the tests [45], by pre-specifying diagnostic goals that can be modified at a local level to suit organizational differences.

Third, reports should describe the care pathways actually followed during the trial, delineating decisions according to the test results and not simply aggregated by study arm. This will be particularly important in pragmatic trials to describe any deviations from the pathway. Embedding process evaluations that measure how interventions are actually administered within a trial [32] may help conceptualize the way in which tests are being used.

We would highlight the need for research on methods of reporting care pathways, variability of care pathways in different settings, and barriers to changing the pathways. Particularly there needs to be an assessment of the information that needs to be reported in a care pathway description to enable its replication in a new setting, and the degree of variability that would be acceptable in pragmatic trials.

### Strengths and limitations

Our study is the first to systematically identify an unselected group of test-treatment RCTs, and assess the quality of intervention reporting. Our cohort includes diverse test-treat interventions, conducted in a wide range of medical settings and specialties. Reporting judgements have been made in duplicate using a standardized extraction tool and disagreements discussed.

We chose to assess whether any detail was mentioned, and not to assess whether the description was adequate to allow the intervention to be replicated (as recommended by the TIDieR checklist), or whether specific features (such as care-provider skill and experience [46]) were detailed. This decision was made to ensure our assessments were objective and because of the challenge in identifying experts able to make judgements across the wide range of settings and specialties in the cohort. Even fewer studies than we report are likely to have reported interventions with adequate detail to allow their replication, including information adequate to establish the appropriateness of care-provider skill used.

The scant reporting we have found certainly reflects inadequate reporting of test-treatment interventions, however it is important that future research investigates the extent to which this may be caused by inadequate trial conduct. This could be achieved by contacting trial authors.

Our trials are from a cohort we have previously reported [23, 29] and were published from 2004 to 7, after publication of the CONSORT 2001 statement but before the CONSORT 2010 and SPIRIT guidelines. It is possible that the reporting of some aspects of the methodology of trials has improved over recent years, particularly given the publication of guidelines for reporting and conducting diagnostic accuracy studies; however any such improvement in describing test-treatment interventions is unlikely to be dramatic since neither CONSORT 2010, STARD 2015, nor any other published standards have addressed test-treatment strategies. Since our sample of trials was ascertained from searching CENTRAL, which indexes relevant studies found in MEDLINE, EMBASE and other specialist registers, it is possible that we have missed eligible test-treatment trials not indexed in these resources. It is arguable, however, that such trials would have changed our findings considerably since in order to have escaped detection by our search of the major databases they are likely to have been more poorly reported [29].

## Conclusions

This review identifies a clear need for improvement in the reporting of interventions in test-treatment trials. Descriptions were so poor that most trial reports we examined are of limited use: their results cannot be interpreted since it is unclear which tests are being evaluated, how these tests are used to inform management, or how treatments are subsequently administered. Since these details are needed to reproduce interventions as safely and effectively as claimed, published trial reports are currently unlikely to enable users to translate interventions into practice. The failure in some pragmatic trials to define how tests should be interpreted and used to direct treatment decisions risks trials failing to demonstrate intended effects due to inadequate implementation, rather than true ineffectiveness.

## Abbreviations

AMO: Amoxicillin
CENTRAL: Cochrane Central Register of Controlled Trials
CONSORT: Consolidated Standards of Reporting Trials
CT: Computed tomography
EMBASE: Excerpta Medica dataBASE
ESO: Esomeprazole
LBP: Lower back pain
LEV: Levofloxacin
MEDLINE: Medical Literature Analysis and Retrieval System Online
MRC: Medical Research Council
RCT: Randomised controlled trial
SPIRIT: Standard Protocol Items: Recommendations for Interventional Trials
STARD: Standards for Reporting of Diagnostic Accuracy
TIDieR: Template for Intervention Description and Replication
